# Co-Treatment of Purified Annatto Oil (*Bixa orellana* L.) and Its Granules (Chronic^®^) Improves the Blood Lipid Profile and Bone Protective Effects of Testosterone in the Orchiectomy-Induced Osteoporosis in Wistar Rats

**DOI:** 10.3390/molecules26164720

**Published:** 2021-08-04

**Authors:** Arlindo César Matias Pereira, Helison de Oliveira Carvalho, Danna Emanuelle Santos Gonçalves, Karyny Roberta Tavares Picanço, Abrahão Victor Tavares de Lima Teixeira dos Santos, Heitor Ribeiro da Silva, Francinaldo Sarges Braga, Roberto Messias Bezerra, Alessandro de Sousa Nunes, Maira Tiyomi Sacata Tongo Nazima, Júlia Gomes Cerqueira, Talisson Taglialegna, Janayra Maris Teixeira, José Carlos Tavares Carvalho

**Affiliations:** 1 Laboratório de Pesquisa em Fármacos, Departamento de Ciências Biológicas e da Saúde, Colegiado de Farmácia, Universidade Federal do Amapá, Macapá 68902-280, AP, Brazil; arlindo.bio@outlook.com (A.C.M.P.); helison_farma@hotmail.com (H.d.O.C.); danna.fernandes@hotmail.com (D.E.S.G.); karynnylovely@gmail.com (K.R.T.P.); abrahaolima28@gmail.com (A.V.T.d.L.T.d.S.); heitor_ribeiro_silva@hotmail.com (H.R.d.S.); alessandronunes82@yahoo.com.br (A.d.S.N.); mairatongu@gmail.com (M.T.S.T.N.); jrate@usp.br (J.M.T.); 2 Programa de Pós-Graduação em Inovação Farmacêutica, Departamento de Ciências Biológicas e da Saúde, Colegiado de Farmácia, Universidade Federal do Amapá, Macapá 68902-280, AP, Brazil; 3 Laboratório de Absorção Atômica e Bioprospecção, Departamento de Ciências Biológicas e da Saúde, Colegiado de Farmácia, Universidade Federal do Amapá, Macapá 68902-280, AP, Brazil; francinaldo1984@gmail.com (F.S.B.); messias@unifap.br (R.M.B.); 4 Faculdade de Medicina, Universidade Federal de Minas Gerais, Belo Horizonte 31270-901, MG, Brazil; cerqueira.jgc@gmail.com (J.G.C.); talissont@gmail.com (T.T.)

**Keywords:** calcium, androgen deficiency, bone, aging, achiote

## Abstract

This study aimed to evaluate and compare the effects of co-treatment with purified annatto oil (PAO) or its granules (GRA, Chronic^®^) with that of testosterone on the orchiectomy-induced osteoporosis in Wistar rats. After surgery, rats were treated from day 7 until day 45 with testosterone only (TES, 7 mg/kg, IM) or TES + PAO or GRA (200 mg/kg, p.o.). The following parameters were evaluated: food/water intake, weight, HDL, LDL, glucose, triglycerides (TG), total cholesterol (TC), alkaline phosphatase levels, blood phosphorus and calcium contents, femur weight, structure (through scanning electron microscopy), and calcium content (through atomic absorption spectrophotometry). Our results show that orchiectomy could significantly change the blood lipid profile and decrease bone integrity parameters. Testosterone reposition alone could improve some endpoints, including LDL, TC, bone weight, and bone calcium concentration. However, other parameters were not significantly improved. Co-treatment with PAO or GRA improved the blood lipid profile and bone integrity more significantly and improved some endpoints not affected by testosterone reposition alone (such as TG levels and trabeculae sizes). The results suggest that co-treatment with annatto products improved the blood lipid profile and the anti-osteoporosis effects of testosterone. Overall, GRA had better results than PAO.

## 1. Introduction

Osteoporosis is a disease that causes significant morbidity and mortality in the elderly population owing to decreased bone mineral density. It is estimated that 30% of women and 12% of men will eventually have osteoporosis [1]. Despite being more common in women, osteoporosis in men is still a considerable burden on public health, and the costs increase as the population ages [2]. It is estimated that, after 50 years, the risk of osteoporotic fractures in men is between 20% and 25%. In addition, men are more prone than women to disabilities and deaths caused by fracture complications [3]. The disease is generally asymptomatic, but eventually, minor trauma can cause fractures. The leading cause of osteoporosis in men is aging-induced androgen deficiency [4].

Sex steroids’ dysregulation can cause osteoporosis because these compounds are responsible for regulating bone growth and maturation. Several studies on sex steroids focus on the effect of estrogen on bones as it has a well-established role in regulating its metabolism, which is evidenced by bone mass loss after natural menopause [5]. To induce osteoporosis experimentally in animals, an orchiectomy is performed, causing hampered testosterone production. The loss of androgens in rats after orchiectomy leads to a condition similar to that of the disease [6].

Mounting evidence points to the potential of *Bixa orellana* products (annatto) for use against osteoporosis [7,8,9]. Chronic^®^, in turn, is a nutraceutical composed of granules of a standardized annatto powder extract. This plant, whose seeds are a source of geranylgeraniol and tocotrienols (90% δ and 10% γ), is native to South America [10]. Tocotrienols belong to the family of vitamin E and are essential, fat-soluble nutrients found in cells’ membranes, possessing antioxidant, neuroprotective, hypocholesterolemic, anticancer, anti-inflammatory, and bone protective properties [11]. Geranylgeraniol is the major oily compound of annatto, representing 1% of its dry weight [12]. This molecule is an intermediate in vitamins E and K’s biosynthesis and is an essential metabolic derivative in the isoprenoid/cholesterol synthesis pathway. There is some evidence that this compound can enhance testosterone production in testis cells [13].

Deng et al. [14] reported that treatment with γ-tocotrienol at 100 mg/kg subcutaneously once per month for three months significantly protected mice from ovariectomy-induced bone loss, as evidenced by structural parameters, bone metabolic gene expression, and serum biochemical markers. Moreover, Wong et al. [7] tested the effects of different doses of annatto tocotrienol on high-carbohydrate high-fat diet-induced metabolic syndrome and osteoporosis. The treatments improved the femur microstructure, biomechanical strength, remodeling activity, hormonal changes, and inflammatory responses. Other studies can be reviewed in [15,16].

The present study aimed to evaluate the effect of testosterone co-treatment with purified annatto oil (PAO) and its granules (Chronic^®^, GRA) on orchiectomy-induced osteoporosis in Wistar rats. This is the first study assessing the co-treatment of testosterone with annatto products, to the best of our knowledge. Moreover, it is the first report showing the effects of annatto treatment as granules. Our results suggest that the combined treatment of testosterone with annatto products improves the efficacy of the hormone.

## 2. Results

**Figure 1:**
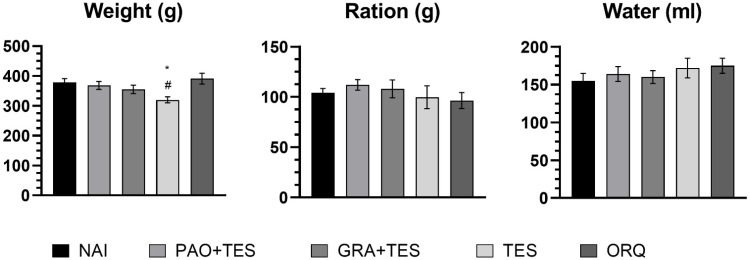
Effect of treatments over 45 days on weight, food intake, and water consumption. NAI: control naïve group; PAO + TES: orchiectomized group treated with purified annatto oil (200 mg/kg, p.o.) + testosterone (7 mg/kg, IM); GRA + TES: orchiectomized group treated with PAO granules (200 mg/kg, p.o., Chronic^®^) + testosterone (7 mg/kg, IM); TES: orchiectomized group treated only with testosterone (7 mg/kg, IM); ORQ: orchiectomized group treated only with water. *: *p* < 0.05 compared with ORQ; #: *p* < 0.05 compared with NAI.

After 45 days, it was observed that the orchiectomized untreated group (ORQ) tended to weigh more, but the difference was not statistically significant compared with the control uncastrated group (NAI). On the other hand, the orchiectomized group treated only with testosterone (TES) had a visible weight decrease (Figure 1). The groups treated with annatto products weighed similar to NAI. No statistically significant differences were observed in food and water intake (Figure 1).

**Figure 2:**
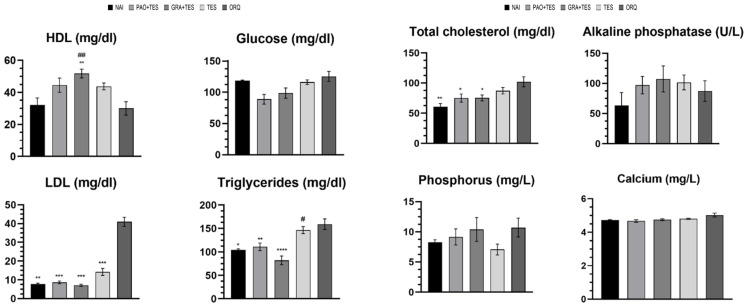
Effect of treatments over 45 days on blood biochemical parameters. NAI: control naïve group; PAO + TES: orchiectomized group treated with purified annatto oil (200 mg/kg, p.o.) + testosterone (7 mg/kg, IM); GRA + TES: orchiectomized group treated with PAO granules (200 mg/kg, p.o., Chronic^®^) + testosterone (7 mg/kg, IM); TES: orchiectomized group treated only with testosterone (7 mg/kg, IM); ORQ: orchiectomized group treated only with water. *: *p* < 0.05; **: *p* < 0.01; ***: *p* < 0.001; ****: *p* < 0.0001 compared with ORQ; #: *p* < 0.05; ##: *p* < 0.01 compared with NAI.

After 45 days, the animals were euthanized and their blood was harvested for biochemical analyses (Figure 2). Except for glucose levels, statistically significant differences were observed in all other metabolic parameters between groups. ORQ had significantly increased triglycerides (TG), total cholesterol (TC) levels, and a massive LDL increase compared with NAI, while HDL was unchanged. Testosterone reposition alone could inhibit LDL and TC increase with values similar to NAI, but TG was still affected. Moreover, TES had HDL values similar to NAI and ORQ.

For the groups co-treated with testosterone and PAO or GRA, there was no statistical difference in LDL, TG, or TC compared with NAI. Moreover, it is observed that co-treatment with annatto products increased HDL levels (the “good cholesterol”), with the highest increase observed in GRA + TES. Overall, GRA had better efficacy in the blood lipid profile than PAO (Figure 2).

Despite not being significantly different, alkaline phosphatase tended to be lower in TES. There were no statistical differences in serum phosphorus and calcium content among groups. ORQ had a slightly higher serum calcium concentration compared with the other groups (Figure 2).

Animals’ femurs were analyzed following blood assessment. This bone was chosen because it is the longest bone and has the highest content of bone mass; it can be easily sectioned for observation on scanning electron microscopy and has previously been used for this purpose elsewhere [9].

**Figure 3:**
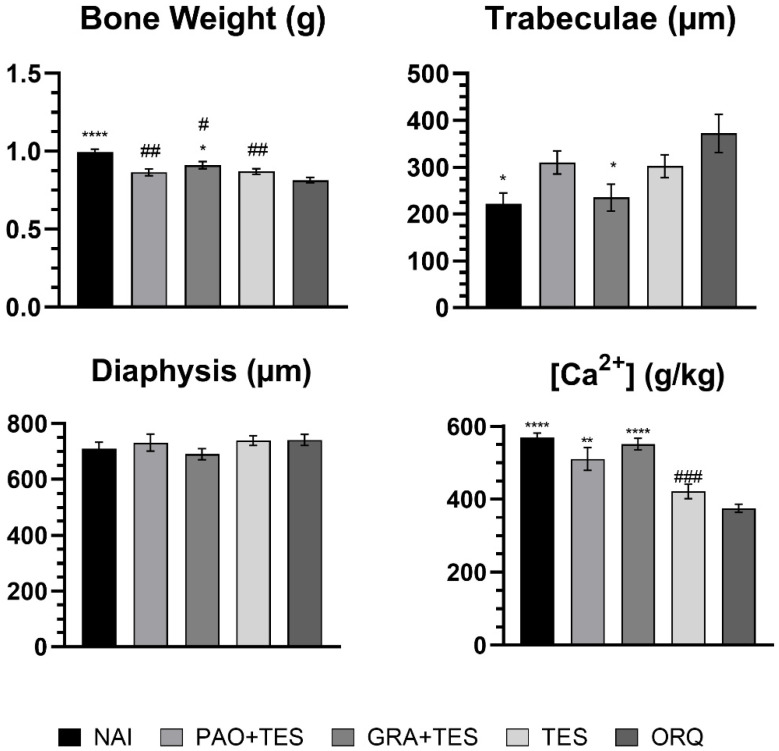
Effect of treatments over 45 days on blood biochemical parameters. NAI: control naïve group; PAO + TES: orchiectomized group treated with purified annatto oil (200 mg/kg, p.o.) + testosterone (7 mg/kg, IM); GRA + TES: orchiectomized group treated with PAO granules (200 mg/kg, p.o., Chronic^®^) + testosterone (7 mg/kg, IM); TES: orchiectomized group treated only with testosterone (7 mg/kg, IM); ORQ: orchiectomized group treated only with water. *: *p* < 0.05; **: *p* < 0.01; ****: *p* < 0.0001 compared with ORQ; #: *p* < 0.05; ##: *p* < 0.01; ###: *p* < 0.001 compared with NAI.

**Figure 4:**
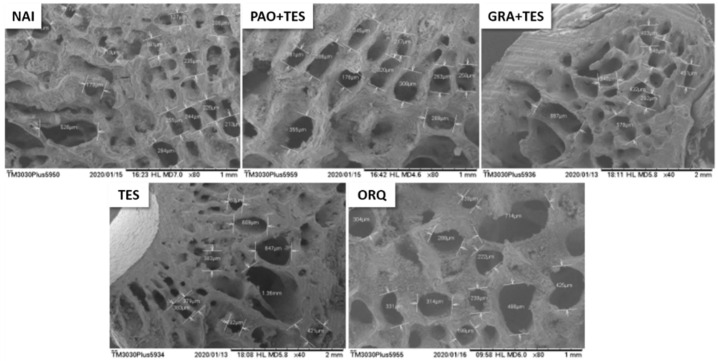
Representative scanning electron microscopy (SEM) pictures from transversal sections of the femur metaphysis with the trabeculae after 45 days of treatment. All the pictures were taken from the same area (in scaled from 1 mm to 2 mm) across the groups to ensure uniformity of the results. Note the more prominent trabeculae in the ORQ group. NAI: control naïve group; PAO + TES: orchiectomized group treated with purified annatto oil (200 mg/kg, p.o.) + testosterone (7 mg/kg, IM); GRA + TES: orchiectomized group treated with PAO granules (200 mg/kg, p.o., Chronic^®^) + testosterone (7 mg/kg, IM); TES: orchiectomized group treated only with testosterone (7 mg/kg, IM); ORQ: orchiectomized group treated only with water.

Statistically significant differences were observed in all endpoints, except for diaphysis size (Figure 3). A huge increase in trabecular bone size was observed in the ORQ group compared with that in the NAI group, evidencing loss of bone density (Figure 3 and Figure 4). Moreover, the ORQ average bone weight and calcium concentration were significantly lower than those of all other groups. Testosterone reposition alone tended to decrease trabeculae sizes compared with those of ORQ, but the means were not significantly different. In the same way, TES had a higher bone weight and bone calcium concentration than ORQ, but without statistical significance.

Interestingly, co-treatment of testosterone with annatto granules (GRA + TES) decreased trabeculae sizes to values similar to those of NAI. Co-treatment with annatto oil (PAO) had smaller trabeculae size values than ORQ, similar to TES, albeit not statistically significant. On the other hand, both PAO + TES and GRA + TES significantly increased bone calcium concentration with values similar to the uncastrated group.

**Figure 5:**
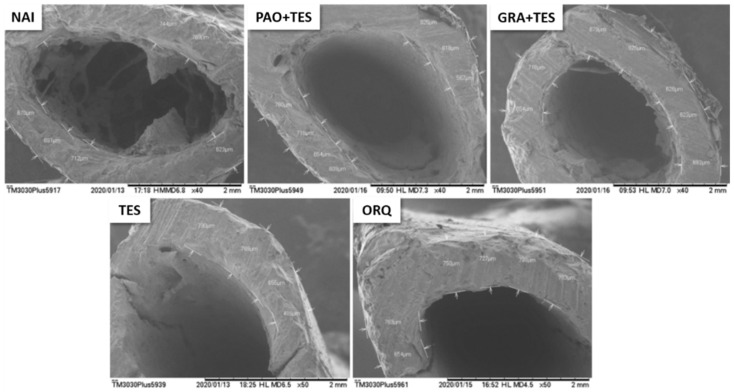
Representative scanning electron microscopy (SEM) of transversal sections of the femur body after 45 days of each group treatment. All the pictures were taken from the same area (in 2 mm scaled) across the groups to ensure uniformity of the results. No statistical divergences were observed among groups. NAI: control naïve group; PAO + TES: orchiectomized group treated with purified annatto oil (200 mg/kg, p.o.) + testosterone (7 mg/kg, IM); GRA + TES: orchiectomized group treated with PAO granules (200 mg/kg, p.o., Chronic^®^) + testosterone (7 mg/kg, IM); TES: orchiectomized group treated only with testosterone (7 mg/kg, IM); ORQ: orchiectomized group treated only with water.

**Figure 6:**
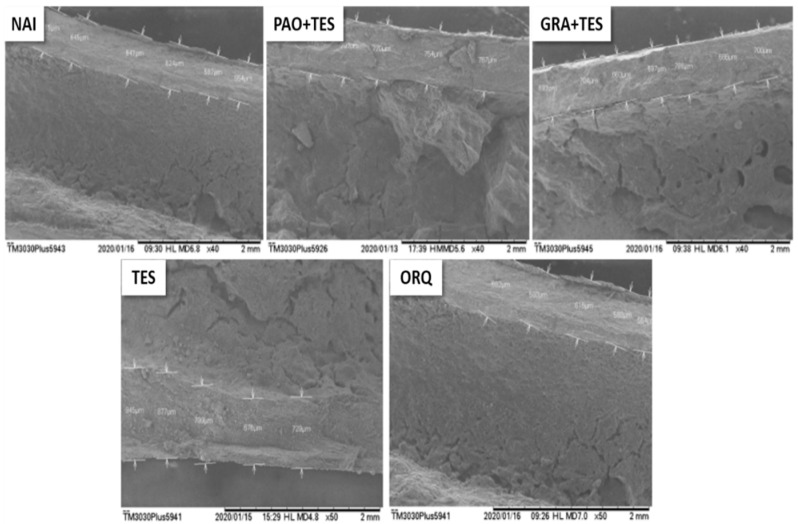
Representative scanning electron microscopy (SEM) of longitudinal sections of the femur body. No statistical divergences were observed among groups. All the pictures were taken from the same area (in 2 mm scaled) across the groups to ensure uniformity of the results. NAI: control naïve group; PAO + TES: orchiectomized group treated with purified annatto oil (200 mg/kg, p.o.) + testosterone (7 mg/kg, IM); GRA + TES: orchiectomized group treated with PAO granules (200 mg/kg, p.o., Chronic^®^) + testosterone (7 mg/kg, IM); TES: orchiectomized group treated only with testosterone (7 mg/kg, IM); ORQ: orchiectomized group treated only with water.

Figure 4, Figure 5 and Figure 6 show animals’ femurs according to their group (visualized through scanning electron microscopy). Figure 4 depicts the transversal sections of the femur metaphysis with the trabeculae. Figure 5 shows the transverse areas of the femur body, and Figure 6 shows its longitudinal sections.

## 3. Discussion

Induction of abnormal food or water intake can be a sign of toxicity. In this study, no significant differences were observed in water consumption and food intake between the treated and control (Figure 1). However, there was a slightly higher weight in ORQ, while in TES, the final weight was significantly lower than NAI. Weight gain is a typical feature of castrated animals and is reported experimentally in several species, including chicken [17], rabbits [18], monkeys [19], and others. It is known that testosterone has an anabolic effect and can increase fat-free mass in hypogonadal patients [20]; however, the general tendency of clinical testosterone therapy is causing whole body weight loss [21], in line with our results.

With respect to metabolic parameters (Figure 2), significantly higher levels were observed in ORQ for LDL, TG, and TC than those of the control NAI group, while HDL was unaffected. Despite considerably improving the blood lipid levels, testosterone alone could not inhibit the TG increase. This is consistent with previous clinical observations where standard testosterone replacement in older men and men with hypopituitarism could decrease LDL and TC levels without affecting TG and HDL [22].

On the other hand, both PAO and GRA co-administered with testosterone could mitigate TG elevation. In fact, in GRA + TES, TG levels were even lower than in the uncastrated group. This is in accordance with Wong et al. [7], who treated Wistar rats fed with a high-carbohydrate high-fat diet with annatto tocotrienols. According to the authors, the treatments (60 and 100 mg/kg) could prevent increased TG, TC, and LDL levels (only the highest dose decreased LDL levels significantly). Conversely, the treatments increased HDL levels, although the difference was not in a statistical manner. Here, both PAO and GRA co-treatment increased HDL levels, but only in GRA + TES was the difference statistically significant (Figure 2).

Although geranylgeraniol is part of the mevalonate pathway (with cholesterol as a product), treatment with the compound could improve the serum lipid profile. This is because both geranylgeraniol and tocotrienols can reduce the rate of cholesterol and triglycerides biosynthesis through negative regulation of HMG-CoA reductase (HGMR) [23,24]. Consequently, they can reduce the risk of developing coronary heart disease. In line with this, treatments with δ-tocotrienol in hypercholesterolemic patients are reported to decrease LDL, TG, and TC levels significantly [25]. Although HMGR inhibition is the same effect caused by statins, the myotoxicity—one of the side effects of these agents [26]—would not happen as it is caused by geranylgeraniol depletion, and this molecule is continuously administered.

Sex steroid hormones are essential for maintaining bone health. Testosterone can act directly on androgen receptors (ARs) or be converted to 5α-dihydrotestosterone in peripheral tissues—which has high affinity for ARs. Studies have shown that testosterone and 5α-dihydrotestosterone stimulate the proliferation and differentiation of osteoblasts and suppress their apoptosis by interacting with their ARs. In addition, stimulation of osteoclast ARs seems to inhibit bone resorption. Moreover, testosterone can be converted to estrogens through aromatization, which can activate estrogen receptors, involved in the anabolic effect of bone surfaces. Orchiectomy causes decreased levels of androgens, resulting in the proliferation and activation of osteoclasts, which lead to bone loss [4].

Alkaline phosphatase (AP) is an enzyme found on bone cells. However, other isoforms of this enzyme can be found in other tissues, such as the liver. Abnormalities in serum AP levels can be an indicator of bone disease, hence it was evaluated. Pelger et al. [27] reported that 87% of patients who underwent orchiectomy had increased AP activity 2 to 4 weeks after the surgery. In accordance, Morote et al. [28] reported that androgen deprivation could significantly increase the levels of bone AP, while in hypogonadal men treated with testosterone, the opposite occurs [29]. Experimentally, this was also observed in rhesus monkeys castrated for three years [19]. As stated by Nagarajan et al. [19], these increased AP levels that occur in androgen deficiency, potentially leading to bone mass loss, can be an indicator of increased osteoclasts activity. Paradoxically, while increased AP can be observed with increased osteoclasts’ activity, it could also be a sign of increased osteoblasts’ activity [6,11,16]. Hence, evaluating AP activity alone may be insufficient to say if its effect is beneficial or detrimental.

Here, there were no statistical differences observed in serum AP levels between the groups (Figure 2). Considering that the test used was not specific for bone AP, and that this enzyme can be found in several different tissues, this could have influenced the results. Other studies have also reported no significant AP changes after orchiectomy in the literature [30]. Although ORQ had a higher serum calcium and phosphorus concentration, the differences were not statistically significant either (Figure 2).

Bone tissue formation occurs through cell proliferation and calcium salts’ deposition, which relies on the nutritional status of the bone, biochemical, and immunological factors. The mevalonate pathway regulates osteoblastogenesis and osteoclastogenesis via prenylation of small guanosine triphosphate-binding proteins (GTPases) in a way that GTPase activation causes bone loss [16]. As both tocotrienols and geranylgeraniol can downregulate this pathway by decreasing HMGR activity, one can hypothesize that PAO and GRA could help to prevent testosterone deficiency-induced bone mass loss. Moreover, there is some preliminary evidence from in vitro studies that geranylgeraniol can enhance steroidogenesis and testosterone production in testis-derived cells by modulating cAMP/PKA signaling [31].

All groups of orchiectomized animals showed a significant decrease in total femur weight compared with that in the NAI group; however, the difference was less significant in treated animals. Notably, GRA + TES had a statistically higher bone mass compared with ORQ (Figure 3). Observing the femur microstructure revealed that the trabeculae in the spongy bone area increased significantly in ORQ (Figure 3 and Figure 4), indicating bone density loss. Testosterone replacement alone and co-treatment with PAO could decrease average trabeculae size compared with ORQ, but the difference was not statistically significant. On the other hand, co-treatment with the granules (GRA + TES) decreased trabeculae sizes in a meaningful manner, with values close to the uncastrated group (Figure 3). There were no significant differences in diaphysis size between groups (Figure 3), either transversally or longitudinally (Figure 5 and Figure 6).

Assessing the bone calcium levels, it is observed that ORQ showed a marked decrease compared with NAI. In TES, the concentrations were higher than in ORQ, but not in a statistically significant manner. Remarkably, both PAO + TES and GRA + TES showed significantly higher calcium concentrations than ORQ and TES (Figure 3). Our results are consistent with previous reports where tocotrienol supplementation improved bone anabolism, preserved Ca^2+^ levels, prevented bone loss, and increased bone matrix deposition, among other benefits [9,14,32,33,34]. The mechanism behind bone homeostasis improvement by tocotrienols is not only due to HMGR inhibition. These compounds also negatively regulate reactive oxygen species and decrease the expression of pro-inflammatory mediators. Collectively, this will lead to the suppression of osteoclasts’ formation and differentiation, improve mineralization, and increase osteoblasts’ differentiation [35,36,37].

Geranylgeraniol can also have a role in the observed bone protection. Besides acting on the mevalonate pathway, exogenous geranylgeraniol can inhibit the formation of osteoclasts via suppression of the receptor activator of NF-κB ligand expression [38]. Moreover, this molecule is involved in membrane localization of intracellular proteins, particularly the small GTP-binding proteins. Disturbances of these pathways can affect cell migration, metabolism, and apoptosis of osteoblasts, fibroblasts, and endothelial cells [39]. The previously mentioned enhancement of testosterone synthesis by geranylgeraniol would provide a direct mechanism in which the treatment could hamper bone loss in a real case of osteoporosis. However, considering that the animals were castrated, unfortunately, this mechanism would not work because of the lack of testis cells.

This research shows a possible way to improve the effects of exogenous testosterone treatment in an androgen deficiency-induced osteoporosis model using annatto products. Considering that the rats were treated for 38 days (45 − 7), and one rat month is considered equivalent to one human year [40], this is a long-term treatment according to this scale. Here, the granules had better results than the purified annatto oil, which could be owing to pharmacokinetic differences between the products. Other authors have reported using delivery systems to increase the compounds’ bioavailability, significantly improving the treatment [41]. As a limitation of this study, we acknowledge that tests including females would also be relevant. Moreover, other osteoporosis models without castration would provide valuable information if the treatment can increase testosterone production in vivo due to geranylgeraniol, in accordance with previous in vitro reports.

## 4. Materials and Methods

### 4.1. Test Material

The samples of purified annatto oil (PAO) and its granules (GRA, Chronic^®^) were kindly provided by Ages Bioactive Compounds Co. (São Paulo, SP, Brazil). The URU200401 batch analysis certificate (12-04/2020, expiration date 22 April 2022) describes the following composition: bixin (1.7%), total tocotrienols (9.59%), and geranylgeraniol (28.32%). The physical-chemical features of GRA and its obtention method are detailed in the patent application Case Number BR 10 2020 015050-2 [42].

### 4.2. Animals and Ethical Aspects

This study followed the recommendations provided by the Universal Declaration of Animal Rights, by the Brazilian College of Animal Experimentation (COBEA), and resolutions of the Brazilian Federal Council of Veterinary Medicine. The study was approved by the Ethics Committee for the Use of Animals in Research (CEUA) of the Federal University of Amapá (UNIFAP) under no. 008-2019.

The Wistar rats used in this study were provided by the Multidisciplinary Center for Biological Research in the Laboratory Animals Science (CEMIB), University of Campinas (UNICAMP). The animals’ age ranged from 21 to 30 days at the beginning of the tests, weighing approximately 350 g. All the animals were acclimatized before the experiments, then kept in polyethylene cages under controlled temperature (~21 °C), with free access to food (standard rodent ration) and water, under 12 h light/dark cycles.

### 4.3. Orchiectomy and Experimental Design

First, the animals were anesthetized with sodium thiopental (45 mg/kg, i.p.), and orchidectomy was performed as described by Idris [43]. From day 7 after surgery until day 45, the animals were treated according to their group. During this period, food and water intake and final weight were assessed. Next, the animals were euthanized to collect blood samples and the right femur.

Five animals per group were used, as this is a common number for biological assays. The doses for PAO and GRA were based on Nazrun et al. [44]. The groups were divided as follows:

1. Naïve control group (NAI): non-orchiectomized animals that received only distilled water (0.5 mL/kg, p.o.);
2. Experimental group (PAO + TES): orchiectomized animals treated with PAO (200 mg/kg, p.o.) + testosterone (7 mg/kg, IM);
3. Experimental group (GRA + TES): orchiectomized animals treated with GRA (200 mg/kg, p.o.) + testosterone (7 mg/kg, IM);
4. Positive control group (TES): orchiectomized animals treated with only testosterone (7 mg/kg, IM);
5. Negative control group (ORQ): orchiectomized animals that received only distilled water (0.5 mL/kg, p.o.).

### 4.4. Biochemical Analyzes

On day 45 after surgery, the animals were anesthetized with sodium thiopental to collect 1.5 mL of blood from the retroorbital plexus. The sample was centrifuged for 10 min at 5000 r/min. The biochemical parameters assessed were glucose, triglycerides (TG), total cholesterol (TC) and fractions (LDL, HDL), alkaline phosphatase (AP), serum phosphorus, and calcium. All tests were performed using Doles^®^ kit (Goiânia, GO, Brazil). Samples were analyzed using the UVmini-1240 UV/VIS spectrophotometer (Shimadzu, Kyoto, Japan), as described by Carvalho et al. [45].

### 4.5. Femur Scanning Electron Microscopy

The femur of the euthanized animals was removed, dehydrated in an oven at 80 °C, and weighed on an electronic analytical balance (Bioprecisa Model, FA-2104N). The femur was sectioned transversally and longitudinally every 0.2 cm, then analyzed through scanning electron microscopy (Hitachi TM3030PLUS) to quantify the trabeculae and diaphysis size. All the pictures were taken from the same area (in scaled from 1 mm to 2 mm) across the groups to ensure uniformity of the results.

### 4.6. Bone Matrix Calcium Quantification

For bone matrix digestion, 250 mg of each sample was placed in a glass tube. Next, 5 mL of digestion solution was added (a mixture of nitric and perchloric acid, 2:1 *v*/*v*) (nitric acid, HNO_3_; 65%, PA, Vetec; Sigma-Aldrich Ltd., São Paulo, SP, Brazil, and perchloric acid, HClO_4_; 70%, PA, ACS, Vetec; Sigma-Aldrich Ltd.). The mixture was heated at 200 °C and became translucent, indicating the complete digestion of the organic matter. Subsequently, the digested samples were transferred to 50 mL volumetric flasks using filter paper. The flask volume was made up to 50 mL using deionized water. The aliquots were transferred to polyethylene bottles and stored at room temperature.

Calcium levels were determined via atomic absorption spectrophotometry (Shimadzu, Tokyo, Japan) with hollow cathode lamps (422.7 nm) and air-acetylene flame. The analytical curve was constructed using a standard solution of pure Ca^2+^ 1000 ppm (Merck 1.09943 Titrisol, St. Louis, MA, USA), according to the methodology described by Palma et al. [46].

### 4.7. Statistical Analysis

First, results were assessed using the Shapiro-Wilk test of normality and passed, hence they were expressed as a mean ± standard deviation. Groups were compared using one-way analysis of variance (ANOVA) followed by the post-hoc Tukey test for multiple comparisons in the case of statistical significance. If standard deviations were statistically different among groups (assessed by Bartlett’s test), Brown-Forsythe ANOVA was performed, followed by Dunnett’s test multiple comparisons in the case of statistical significance. Statistical significance was set at *p* < 0.05, and the statistical program used was GraphPad Prism 9.

## 5. Conclusions

This study compared the testosterone standard treatment with co-treatment of testosterone and two products derived from annatto (*Bixa orellana)*, the purified oil and its granules (Chronic^®^), on orchiectomy-induced osteoporosis in Wistar rats. Our results showed that the co-treatment of testosterone with annatto products could improve the blood lipid profile and the anti-osteoporosis effect of testosterone. Overall, the granules of annatto exhibited superior results compared with those exhibited by the oil, which could be due to pharmacokinetic differences.

## 6. Patents

A patent deposit resulting from this work was requested at the National Patent and Innovation Institute of Brazil (INPI), with the number BR-102021001428-8.

## Data Availability

Data is contained within the article.

## Sample Availability

Samples of the compounds are available from the authors.
